# Supplementary material for: Tradeoffs in demographic mechanisms underlie differences in species abundance and stability
Source: Nat Commun. 2018 Nov 28;9:5047. doi: 10.1038/s41467-018-07535-w (PMC6261946; doi:10.1038/s41467-018-07535-w)
Supplement: Supplementary file 1 — Supplementary Information [file 41467_2018_7535_MOESM1_ESM.pdf]

Supplemental Information for Hallett et al. Tradeoffs in demographic mechanisms underlie differences in species abundance and stability

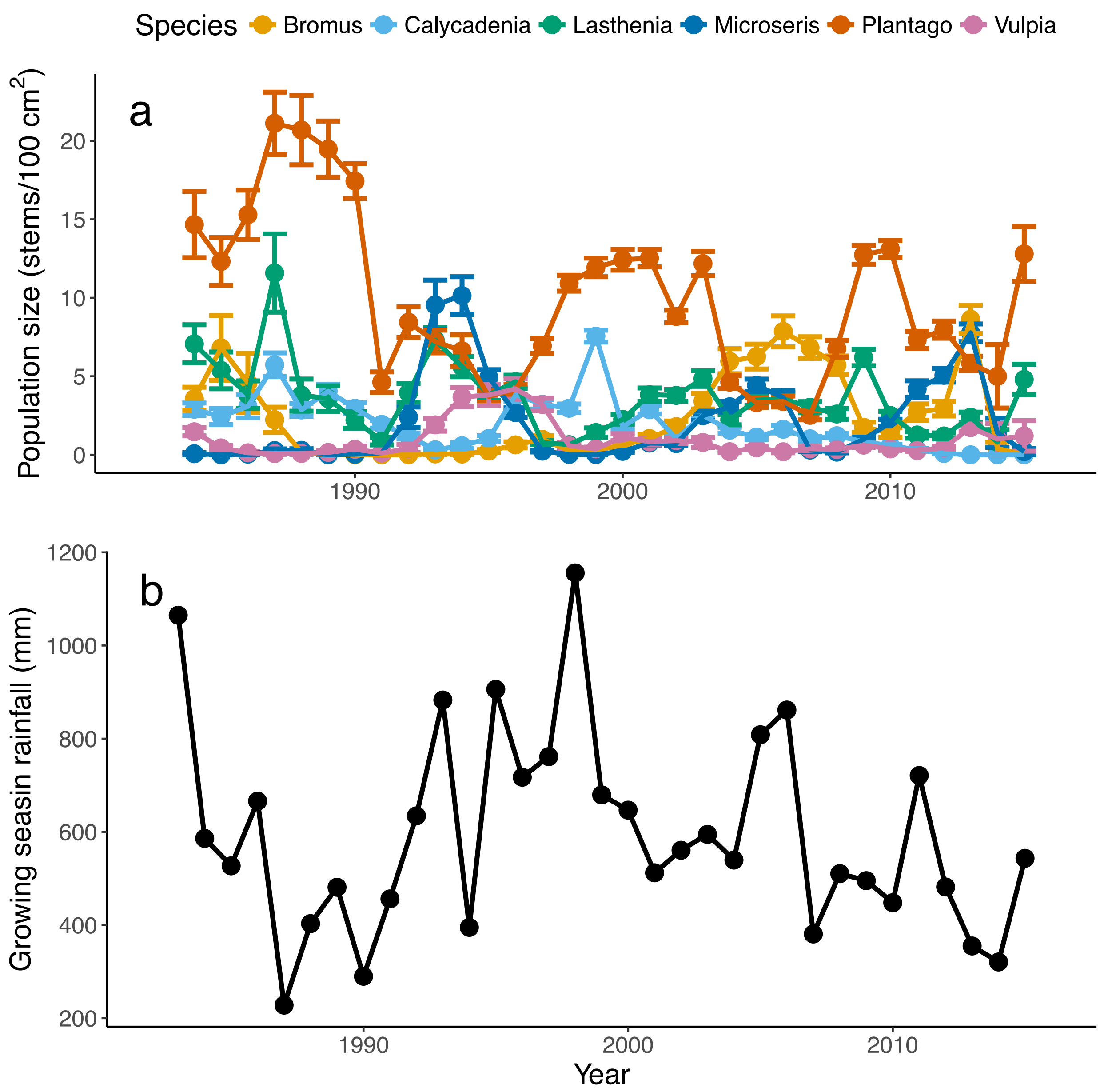

Supplementary Figure 1. a, Mean annual population size of the six focal annual plants species from 1983-2015 (±s.e.m., *n* increases from 30 to 150 over time). **b,** Growing season rainfall at the Jasper Ridge Biological Preserve (Santa Mateo County, CA) from 1983-2015; the growing season occurs between September-April.

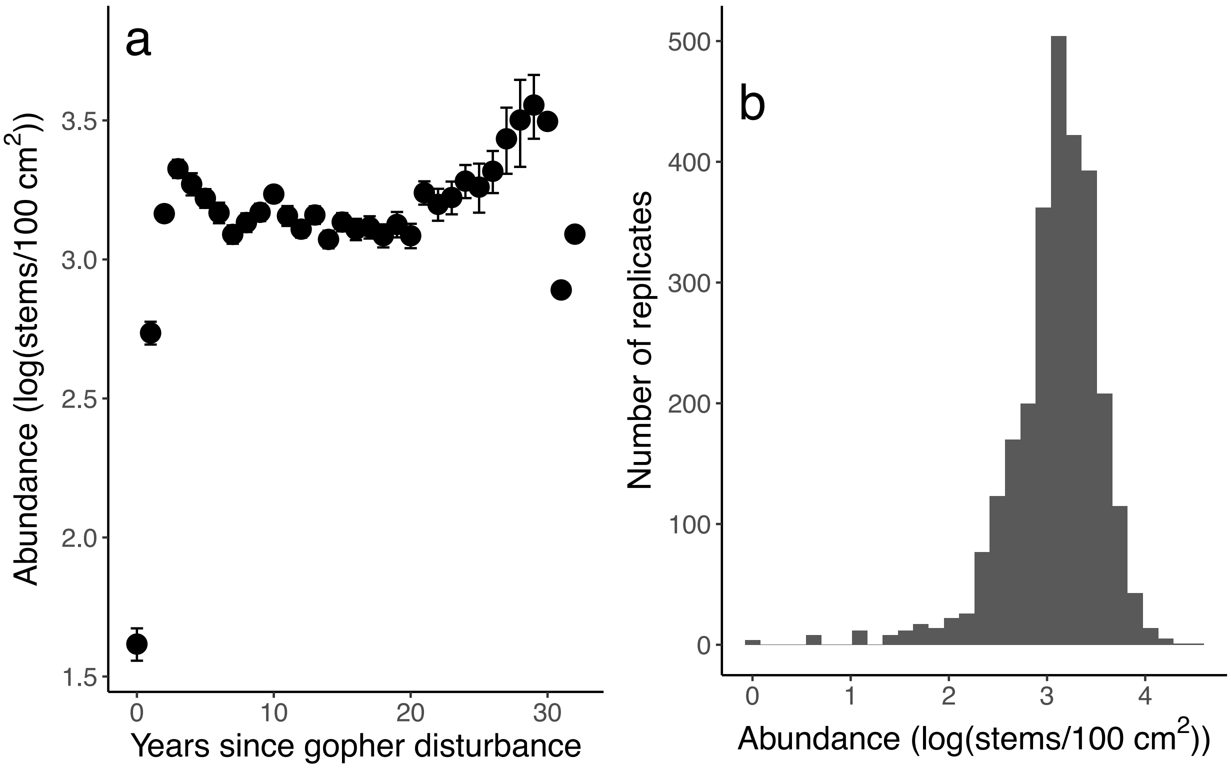

Supplementary Figure 2. a, Total abundance (stem density) of all species in relation to the number of years since a plot experienced gopher disturbance (i.e., gopher disturbance occurs in year 0) (±s.e.m.). Replication is high ($n > 50$ plots/time point) from years 0 through 20, but decreases substantially for longer time points. Our population models only include replicates that did not experience gopher disturbance in the current or previous year (i.e., time points >1). **b,** Histogram of total abundance (stem density) for replicates included in our population models (time points >1).

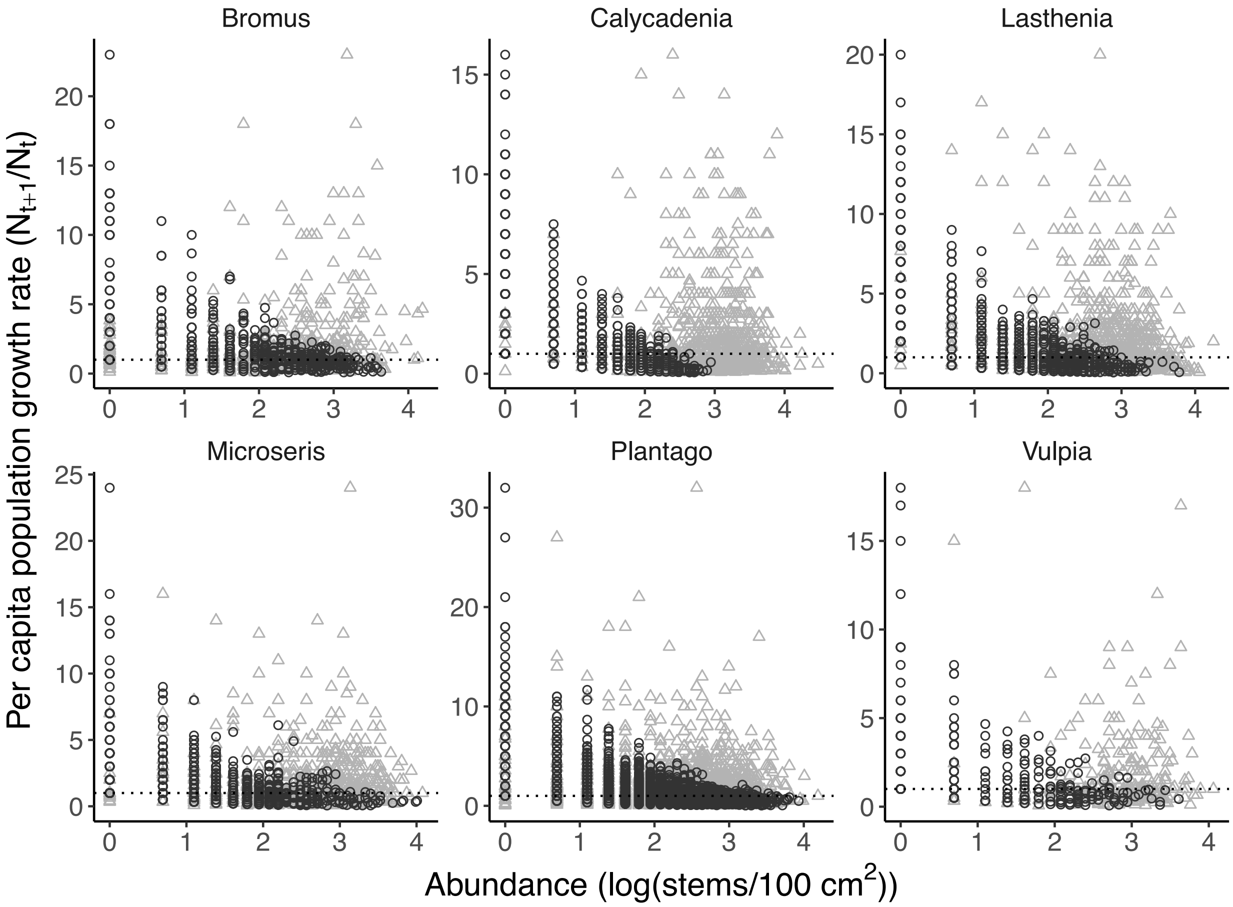

Supplementary Figure 3. Per capita population growth rate (calculated as the stem count in time *t*+1 divided by the stem count in time *t*) in relation conspecific (dark circles) and summed heterospecific (light triangles) abundances at time *t*.

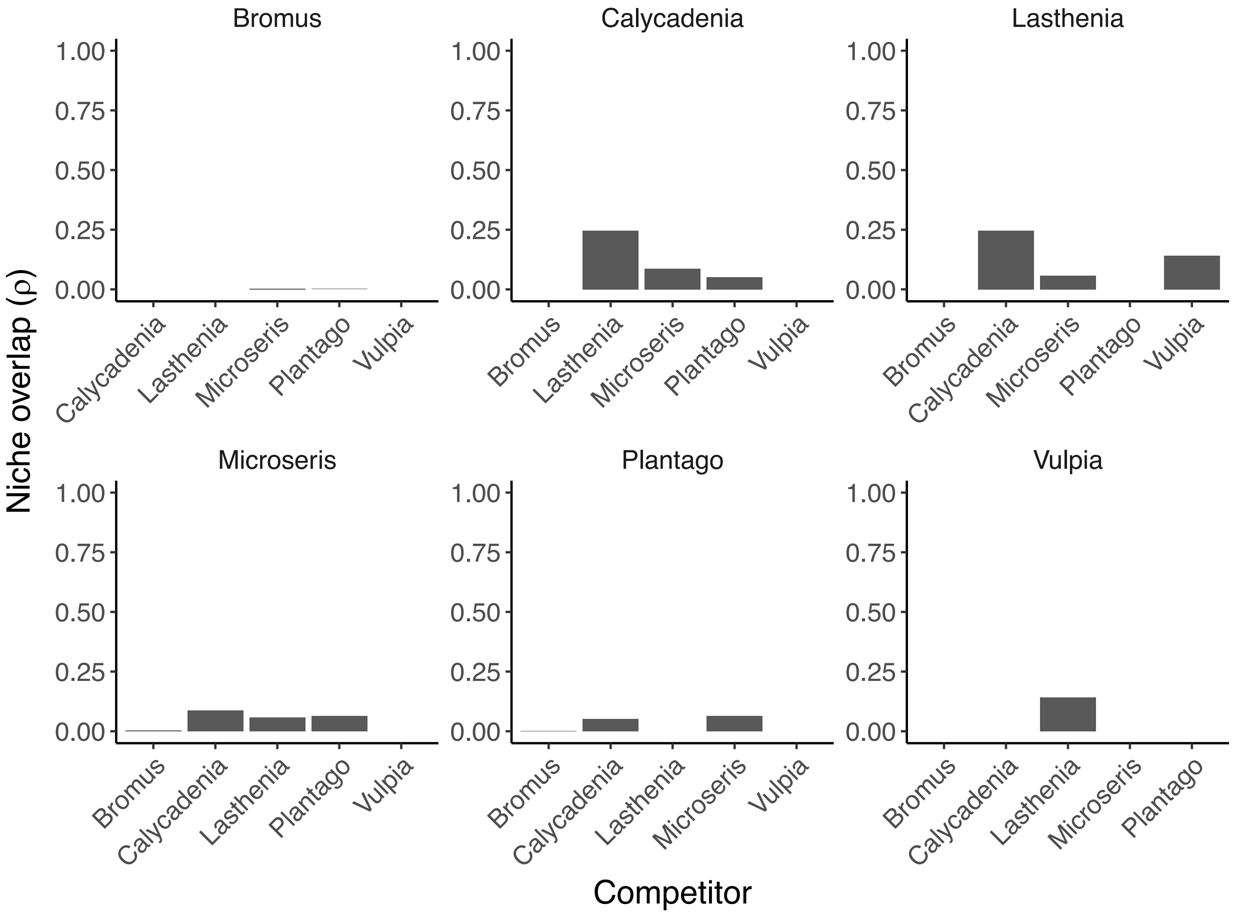

Supplementary Figure 4. Niche overlap between each focal species and five heterospecific competitors. Niche overlaps ranges from 0 (no overlap) to 1 (complete overlap).

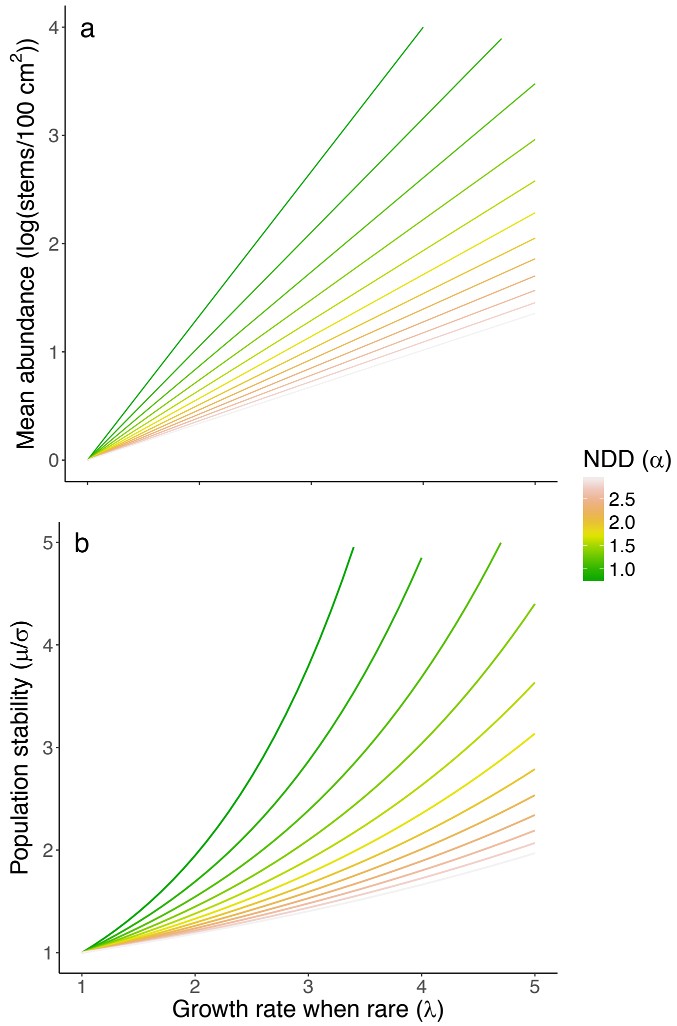

Supplementary Figure 5. a, Predicted effect of species' growth rates when rare (GRWR λ) and negative density-dependence (NDD α) on equilibrium population size. **b**, Predicted effect of GRWR and NDD on population stability.

Supplementary Table 1. Species observed at the study site from 1983-2015, their functional group, and their mean and maximum abundances across the entire site and sampling period. *Denotes the focal species for analyses.

Species	Functional group	Mean abundance (stem/100 cm^2)	Maximum abundance (stem/100 cm^2)
**Plantago erecta*	annual forb	7.46	51
**Lasthenia californica*	annual forb	2.62	44
**Bromus hordeaceus*	annual grass	2.28	38
**Microseris douglasii*	annual forb	1.91	55
**Calycadenia multiglandulosa*	annual forb	1.48	19
**Vulpia microstachys*	annual grass	1.11	37
Hesperevax sparsiflora	annual forb	0.65	36
Lotus wrangelianus	annual legume	0.36	8
Layia platyglossa	annual forb	0.31	14
Brodiaea sp.	geophyte	0.31	12
Castilleja densiflora	annual forb	0.29	14
Micropus californicus	annual forb	0.26	26
Agoseris heterophylla	annual forb	0.24	15
Hemizonia congesta	annual forb	0.19	8
Crassula connata	annual forb	0.09	25
Astragalus gambelianus	annual legume	0.09	7
Festuca perennis	annual grass	0.04	8
Elymus multisetus	perennial grass	0.03	3
Epilobium brachycarpum	annual forb	0.02	3
Gilia clivorum	annual forb	0.02	5
Trifolium sp.	annual legume	0.02	3
Stipa pulchra	perennial grass	0.01	2
Plagiobothrys nothofulvus	annual forb	0.01	5
Calandrinia ciliata	annual forb	0.01	2
Escholtzia californica	perennial forb	0.01	1
Lomatium utriculatum	perennial forb	0.01	1
Chlorogalum pomeridianum	geophyte	0.01	2
Poa secunda	perennial grass	0.004	1
Lepidium nitidum	annual forb	0.001	2
Melica california	perennial grass	0.001	2

Supplementary Table 2. Maximum likelihood tests to compare candidate models that include interspecific competition terms. Models describe the relationship between a species' population size, its potential rate of increase (λ), its degree of negative density dependence (α_{ii}) and its response to growing season rainfall (β), and interspecific competition (α_{ij}). Count values were logged prior to analysis. The best-fit model (averaged across species) is bolded.

Model Structure	AIC (normal)
$N_{i,t+1} = \dfrac{(\lambda_i + \beta_i Rain_{t+1})N_{i,t}}{1 + (\alpha_{ii}N_{i,t} + \sum_1^j \alpha_{ij}N_{j,t})}$	**75432**
$N_{i,t+1} = (\lambda_i + \beta_i Rain_{t+1})e^{-\alpha_{ii}\ln(N_{i,t}+1) - \sum_1^j \ln(N_{t,j}+1)}$	75437
$N_{i,t+1} = (\lambda_i + \beta_i Rain_{t+1})e^{-\alpha_{ii}N_{i,t} - \sum_1^j \alpha_{ij}N_{j,t}}$	75841
$N_{i,t+1} = 1 + (\lambda_i + \beta_i Rain_{t+1})(1 - \alpha_{ii}N_{i,t} - \sum_1^j \alpha_{ij}N_{j,t})$	76289
$N_{i,t+1} = \dfrac{(\lambda_i + \beta_i Rain_{t+1})N_{i,t}}{1 + (N_{i,t})^{\alpha_{ii}} + \sum_1^j (N_{j,t})^{\alpha_{ij}}}$	103358

Supplementary Table 3. Parameter estimates (± s.e.m.) of the best-fit population models that include terms for interspecific competition for the six focal species (see Supplementary Table 2). Parameter estimates include a species' growth rate when rare (λ), its degree of intraspecific density dependence (α_{ii}, indicated by the species' effect on itself), its response to growing season rainfall (β) and its response to interspecific competition (α_{ij}, indicated by other species' effects on the focal species). Count values were logged prior to analysis. Values significant at $P < 0.05$ are bolded.

Species	λ	β	*Bromus*	*Calycadenia*	*Lasthenia*	*Microseris*	*Plantago*	*Vulpia*
Bromus	**3.82 ±0.93**	**0.22 ±0.11**	**1.12 ±0.36**	0.080 ±0.12	0.057 ±0.092	0.071 ±0.097	0.023 ±0.086	0.21 ±0.13
Calycadenia	20.3 ±44.0	1.51 ±3.28	5.90 ±13.02	13.66 ±30.13	0.22 ±0.90	0.64 ±1.89	1.29 ±3.27	0.023 ±0.84
Lasthenia	13.27±10.47	0.049±0.35	1.29 ±1.19	0.45 ±0.55	5.73 ±4.80	1.35 ±1.19	0.81 ±0.80	1.20 ±1.11
Microseris	**5.86 ±2.35**	0.20 ±0.17	0.86 ±0.48	0.35 ±0.30	0.17 ±0.17	2.26 ±1.04	0.030±0.15	**1.75 ±0.84**
Plantago	**7.69 ±1.30**	**-0.19 ±0081**	**0.22 ±0.088**	0.027 ±0.070	**0.015 ±0.071**	**0.77 ±0.17**	**2.66 ±0.51**	**0.13 ±0.087**
Vulpia	**2.07 ±0.66**	0.12 ±0.10	0.03 ±0.10	0.32 ±0.19	-0.107 ±0.089	-0.011 ±0.089	-0.11 ±0.079	**0.72 ±0.32**

Supplementary Table 4. Maximum likelihood tests to compare candidate models describing how a species' population size is affected by its growth rate when rare (λ), its degree of intraspecific density dependence (α) and its response to growing season rainfall (β). Count values were logged prior to analysis. Predicted equilibrium abundance under average rainfall conditions is reported for each model. The best-fit model (averaged across species) is bolded.

Model structure	Predicted equilibrium abundance	AIC
$N_{t+1} = \dfrac{(\lambda + \beta Rain_{t+1})N_t}{1+(\alpha N_t)}$	$(\lambda - 1)/\alpha$	**14656**
$N_{t+1} = (\lambda + \beta Rain_{t+1})e^{-\alpha \ln(N_t+1)}$	$e^{\ln(\lambda)/\alpha - 1}$	14659
$N_{t+1} = \dfrac{(\lambda + \beta Rain_{t+1})N_t}{1+(N_t)^{\alpha}}$	$(\lambda - 1)^{1/\alpha}$	14660
$N_{t+1} = (\lambda + \beta Rain_{t+1})e^{-\alpha N_t}$	*Solve numerically*	14739
$N_{t+1} = 1 + (\lambda + \beta Rain_{t+1})(1 - \alpha N_t)$	$(1+\lambda)/(1+\alpha\lambda)$	14818

Supplementary Table 5. Parameter estimates (± s.e.m.) for the best fit model that did not directly incorporate interspecific competition (Supplementary Table 3). Parameter estimates include a species' growth rate when rare (λ), its degree of intraspecific negative density-dependence (α), and its response to growing season rainfall (β). Terms significant at $P < 0.05$ are bolded.

Species	λ	α	β
Bromus	**2.96 ±0.44**	**0.83 ±0.19**	0.07 ±0.07
Calycadenia	**3.66 ±1.07**	**2.44 ±0.92**	**0.35 ±0.16**
Lasthenia	**3.49 ±0.65**	**1.62 ±0.41**	-0.07 ±0.09
Microseris	**2.87 ±0.45**	**1.01 ±0.24**	-0.04 ±0.07
Plantago	**4.04 ±0.35**	**1.34 ±0.16**	**-0.09 ±0.04**
Vulpia	**2.25 ±0.55**	**0.75 ±0.32**	0.11 ±0.12

Supplementary Methods

Ecological Assumptions

Minimal seed bank carryover

Our model assumes that there is minimal seed bank carryover, and consequently that recruitment is a sole product of seed input from the year before. Below we outline evidence from which we based this assumption.

1) Germination from soil collected at different times in the year. Hobbs and Mooney collected 25 soil samples from 4 different soil depths (0-1 cm, 1-2 cm, 5-6 cm, 10-11 cm) in 2 month intervals from Oct 1983-Dec 1984 at our site, and measured germination from these samples in a greenhouse over 8-week periods[1]. Each of our focal species were well-represented in aboveground community composition over that time period. They observed very high rates of seed germination from soils collected in the summer and early autumn, but virtually no germination for soils collected in the spring (i.e., after recruitment and before seed set). This indicates that most seeds in the soil germination in the first year, and that carryover is minimal.

2) Germination of collected seeds across different environmental conditions. Gulmon collected seeds of each of our focal species from our site and grew them under four different rainfall treatments (with rainfall commencing in mid September versus mid October, November, or December, and thereafter maintained at prevailing weather conditions), in addition to growing seeds under different levels of topsoil and litter[2]. She found that, except for when rainfall began abnormally early (i.e., September), cumulative germination across species was generally high, suggesting that most seeds germinate in the first year regardless of environmental conditions. Specifically, across rainfall conditions she observed germination rates between 90 and 100% for *Plantago*, *Bromus*

and *Vulpia* and between 70 and 90% for *Microseris* and *Lasthenia*). *Calycadenia* was the exception, with cumulative germination rates dependent on time (i.e., low germination rates following late first-germinating rains). Most species exhibited high germination rates regardless soil depth, suggesting that most seeds in the topsoil will germinate in the concurrent year.

Strong niche differentiation

Ecological communities are shaped by processes that select for species to be similar, such as environmental filtering, and processes that select for species to be dissimilar, such as competition ultimately resulting in niche differentiation. Species coexistence can be a product of both these processes, such that coexistence can be enhanced if species have similar average growth rates (equalizing forces) and can be maintained if species occupy different niches (stabilizing forces)[3]. For communities characterized by strong niche differentiation (indicated by stronger intra- than inter- specific competition), growth rate when rate (GRWR, λ_i) and intraspecific negative density-dependence (NDD, α_{ii}) should be the primary determinates of a species' average abundance, and interspecific competition should be relatively diffuse in relation to heterospecific competitors, particularly if total stem density is reasonably consistent over time (as it is in our system; Supplementary Figure 2).

Previous research in our system suggests it is shaped by strong niche differentiation[4], which supports a focus on GRWR and NDD for understanding abundance patterns. To examine this assumption, we first visualized per capita population growth rate (calculated as the change in stem count between time *t* and time *t* +1) in relation conspecific and summed heterospecific abundances at time *t*. This visualization

highlights that growth rates consistently decrease with conspecific abundance but not summed heterospecific abundance for all species in our analyses (Supplementary Figure 3). In other words, intraspecific density dependence appears to be a stronger determinant of species' growth rates than total density dependence.

Second, we used our population models to parameterize the degree of niche overlap between species. We calculated niche overlap as:

$$\rho = \sqrt{\frac{\alpha_{ij}}{\alpha_{jj}}\frac{\alpha_{ji}}{\alpha_{ii}}}$$

where complete niche differentiation is indicated by $\rho = 0$, and complete niche overlap is indicated by $\rho = 1$[5,6]. We calculated ρ for every pairwise species comparison for the top two models; in all instances niche overlap was minimal ($\rho \leq 0.25$ for every pairwise species comparison; Supplementary Figure 4).

Supplementary References

1. Hobbs, R. & Mooney, H. Community and population-dynamics of serpentine grassland annuals in relation to gopher disturbance. *Oecologia* **67,** 342–351 (1985).
2. Gulmon, S. L. Patterns of seed germination in Californian serpentine grassland species. *Oecologia* **89,** 27–31 (1992).
3. Chesson, P. Mechanisms of maintenance of species diversity. *Annu. Rev. Ecol. Syst.* 343–366 (2000).
4. Levine, J. M. & HilleRisLambers, J. The importance of niches for the maintenance of species diversity. *Nature* **461,** 254–257 (2009).
5. Godoy, O. & Levine, J. M. Phenology effects on invasion success: insights from coupling field experiments to coexistence theory. *Ecology* **95,** 726–736 (2014).

6. Kraft, N. J. B., Godoy, O. & Levine, J. M. Plant functional traits and the multidimensional nature of species coexistence. *Proc. Natl. Acad. Sci.* **112,** 797–802 (2015).
